# Androgen metabolism in prostate cancer: recent advances

**DOI:** 10.1210/endocr/bqag021

**Published:** 2026-02-24

**Authors:** Nima Sharifi

**Affiliations:** Desai Sethi Urology Institute, University of Miami Miller School of Medicine, Miami, FL 33136, USA; Sylvester Comprehensive Cancer Center, University of Miami Miller School of Medicine, Miami, FL 33136, USA

**Keywords:** androgens, metabolism, prostate cancer, steroids, enzymes

## Abstract

Androgen biosynthesis is physiologically necessary for generating the principal stimulus for androgen receptor (AR) signaling and thus plays an essential role for development of the normal prostate, prostate cancer growth, and the development of resistance to hormonal therapies. Testosterone and dihydrotestosterone are both potent endogenous androgens that stimulate AR signaling. While the role of gonadal androgens in stimulating prostate cancer progression has been recognized for over 80 years, the appreciation for nongonadal precursor steroids in prostate cancer has been more limited in duration of time, attention, and focus in the field. Nevertheless, the very clearly established role of nongonadal androgens in enabling prostate cancer progression, especially in the absence of gonadal testosterone, frames the essentiality of androgen metabolic processes for dictating prostate cancer clinical behavior. Here, the role of androgen metabolism in prostate cancer is reviewed, particularly within the context of hormonal therapy and hormone therapy resistance, and with emphasis on recent advances.

Androgens and the androgen receptor (AR) comprise a signaling pathway that is essential for development of the normal prostate and for prostate cancer progression. All androgens are synthesized from cholesterol as the initial substrate. In humans, the major physiologic sources of androgens are the testes and the adrenal glands. The dependence of prostate cancer growth on testicular testosterone (T) was recognized over 80 years ago and led to the use of androgen deprivation therapy (ADT) with medical or surgical castration as the upfront systemic treatment for metastatic prostate cancer (1). The vast majority of prostate cancers respond to ADT (2, 3). However, almost all tumors subsequently eventually progress to a state that is termed castration-resistant prostate cancer (CRPC) (4). These progressing tumors switch from a predominant dependence on gonadal T to their use of nongonadal androgens, thought largely to come from adrenal androgen precursors, which are metabolically converted in tumor tissue to potent androgens (5). This resistant state in particular shed a bright spotlight on the essential importance of androgen metabolism in prostate cancer physiology and resistance to hormonal therapy.

## Evolution of hormonal therapies in prostate cancer

At its core, there are 2 approaches for hormonal therapy of prostate cancer (6). The first approach uses methods that suppress the production of androgens that bind and stimulate the AR. The second uses methodologies that directly and competitively bind AR to suppress AR signaling. In the first approach, which entails blockade of androgen production, an understanding of the sources of androgen production is essential. Clearly, testicular production of T is the major androgen source in adult males for stimulation of androgen signaling. While T binds AR, it is also converted in prostatic tissue by steroid-5α-reductase (SRD5A) to the more potent 5α-dihydrotestosterone (DHT) (7). The depletion of gonadal T in circulation leads to a loss of DHT generation and ultimately suppression of AR activity in prostatic tissues. ADT is administered by either surgical castration (orchiectomy) or via gonadotropin-releasing hormone (GnRH) agonists or GnRH antagonists.

In the absence of gonadal T, adrenal precursors are the major alternative androgens for prostate cancer (8). Inhibition of adrenal androgen biosynthesis was previously achieved with ketoconazole, which blocks CYP17A1 but is a problematic drug because it also blocks other cytochrome p450 enzymes. Subsequently, abiraterone was developed as a more potent and specific inhibitor of CYP17A1. Abiraterone is part of the standard-of-care armamentarium today and also considered an androgen receptor signaling inhibitor (ARSI) (9). Pharmacologic inhibition of CYP11A1, the very first enzyme in steroid biosynthesis, is also undergoing active clinical development in prostate cancer (10).

The second approach, of directly targeting the AR, historically used steroidal inhibitors (cyproterone), followed by the development of nonsteroidal inhibitors (flutamide, nilutamide, bicalutamide) (11), and subsequently the current generation of more potent AR antagonists (enzalutamide, apalutamide, darolutamide) (2). The current generation of drugs are also considered ARSIs (9).

The other important development for the evolution of hormonal therapies in prostate cancer is the time and timing of the use of ARSIs. Abiraterone and enzalutamide first demonstrated their survival benefit when used in men with metastatic CRPC after the use of docetaxel chemotherapy (12, 13). Subsequently, a series of phase 3 studies were performed in progressively forward-moving clinical states of disease and has led to the demonstration of an even greater clinical benefit in metastatic prostate cancer when abiraterone or enzalutamide is used upfront along with ADT in men not previously treated with systemic therapy, instead of awaiting resistance to ADT (14-21). Together, these clinical trials support the contention that earlier and intensified hormonal therapy is clinically superior to incremental and serial addition of hormonal therapies.

## Essential biochemical modifications of steroids and enzymes

**Figure 1:**
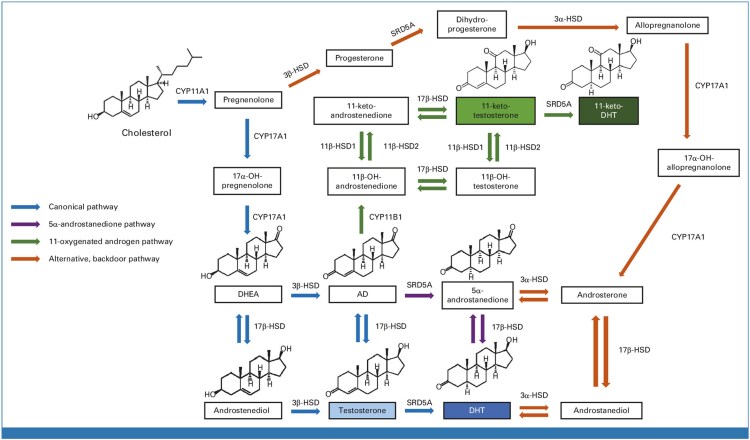
A simplified diagram of some of the major pathways of androgen biosynthesis in prostate cancer. The pathways depicted include the canonical pathway of DHT synthesis that utilizes T as an intermediate metabolite (blue), and the 5α-androstanedione pathway (purple), which occurs from adrenal precursor steroids. The pathways of 11-oxygenated androgens are also shown (green), as is an alternative backdoor pathway (orange). 3βHSD is 3β-hydroxysteroid dehydrogenase-1; 3αHSD is 3α-hydroxysteroid dehydrogenase; 17βHSD is 17β-hydroxysteroid dehydrogenase. Not shown is the CYP17A1-independent pathway that utilizes CYP51A1. From Dai, et al *J Clin Oncol* 2023.

Cholesterol serves as the origin of all steroids (Fig. 1) (22). The conversion of cholesterol to androgens requires the stepwise processing and shortening of the cholesterol side chain while maintaining the essential 4-ring structure (23). The first enzyme in this process is CYP11A1 (also known as side chain cleavage enzyme), which takes 6 carbons off the cholesterol side chain to make 21-carbon pregnenolone. This step is necessary to make all steroid hormones, including glucocorticoids, mineralocorticoids, androgens, and estrogens. The next enzyme is CYP17A1, which catalyzes 2 reactions. First, CYP17A1 hydroxylates C17 and it next cleaves the C17-C20 bond to make 19-carbon dehydroepiandrosterone (DHEA) (24).

DHEA (in free and sulfated forms) is synthesized in the human adrenal reticularis and is the most abundant steroid in circulation in normal physiology. The DHEA sulfate is far more abundant, while DHEA itself is much lower and comparable in concentration to T in men. The 3β-OH, Δ^5^-structure of the A and B rings of cholesterol are preserved as it is metabolized to DHEA. It turns out that this part of the steroid structure holds the key to an essential chemical switch that enables conversion to biologically active androgens and genetically controls prostate cancer clinical outcomes driven by androgen metabolism (25). In peripheral tissues, including prostate cancer, the enzyme 3β-hydroxysteroid dehydrogenase 1 (3βHSD1; encoded by *HSD3B1*) oxidizes 3β-OH to 3-keto and isomerizes the double bond from between carbons 5 and 6 to between carbons 4 and 5 (26, 27). Thus, 3βHSD1 converts DHEA to Δ^4^-androstenedione (AD), which is then converted to DHT via 2 enzymatic steps. AD can be converted first to T and then 5α-reduced to DHT. Alternatively, AD is first 5α-reduced to 5α-androstanedione, which is then converted to DHT (28). Parenthetically, 3βHSD1 also catalyzes the same steroid A/B ring transformations for other steroids, including conversion from pregnenolone to progesterone.

More generally, the 3-keto, Δ^4^-structure of AD and T allow them both to either undergo 5α-reduction by SRD5A enzymes, or become substrates for aromatase, for the generation of estrone and estradiol, respectively. As such, the 3-keto, Δ^4^-structure of AD and T can be viewed as a chemically encoded pivot point that dictates 5α-reduction to the most potent androgen (DHT) and AR stimulation vs aromatization to estrogens and estrogen receptor stimulation (29, 30). Further, studies on the biochemical regulation of the immediate upstream step for the generation of 3-keto, Δ^4^-structure sex steroid precursors, coupled with multiple studies on clinical outcomes, show that essentiality of regulation by germline variability in 3βHSD1 (31).

Carbon 17 serves as an important point of regulation for androgens. Both T and DHT, biologically active androgens, have a 17β-OH structure. Generally, oxidation, that is, conversion from 17β-OH → 17-keto, ablates the ability to bind the AR (32). This turns out to be important both for the inactivation of T and DHT once generated, as well as the biosynthesis of T and DHT from precursor steroids. A major example of an enzyme that regulates the interconversion at carbon 17 is aldo-keto reductase family member C3 (AKR1C3), also known as 17βHSD5, which is upregulated in hormone therapy–resistant prostate cancer and is thought to be the major enzyme that enables conversion of precursor steroids to T and DHT (33, 34).

## 11-oxygenated androgens

While most androgens have been known and characterized for many decades, there is an increasing appreciation for a relatively newly recognized class of androgens. The 11-oxygenated androgens require adrenal CYP11B1 enzyme activity for 11β-hydroxylation of androgens, to make 11β-OH-A4 as the most abundant androgen in this class in the human adrenals (35). Previously, CYP11B1 was more well-known as an enzyme required to catalyze the same carbon 11 modification for the biosynthesis of glucocorticoids. Interestingly, the 11-keto vs 11β-OH conformations of the 11-oxygenated androgens have opposing roles when compared to glucocorticoids. Specifically, for the androgens, the 11-keto conformation is the active form that binds the AR, while for glucocorticoids it is the 11β-OH form that binds the glucocorticoid receptor. Additionally, both the 11-oxygenated androgens and glucocorticoids use a common set of enzymes that interconvert the 11β-OH and 11-keto forms of their respective steroids. Specifically, 11β-HSD1 is the reductive enzyme that converts 11-keto to 11β-OH steroids and 11β-HSD2 is the oxidative enzyme that converts 11β-OH to 11-keto steroids (36). For the 11-oxygenated androgens, 11-keto-testosterone and 11-keto-DHT serve as potent androgens that bind and stimulate the AR (37). Together, the 11-oxygenated androgens add another layer to precursor steroids synthesized by the adrenals that are converted in prostatic and other peripheral tissues to potent AR agonists.

## Adrenal-permissive and adrenal-restrictive *HSD3B1* genetics and clinical outcomes

Emerging evidence suggests that there is a major germline-controlled genetic component of androgen metabolism that also regulates clinical outcomes. The identification of this layer of regulation came at the laboratory bench, with the discovery of cellular metabolic phenotypes that dictate the conversion from DHEA to downstream potent androgens by 3βHSD1 (38). The *HSD3B1* gene that encodes for the 3βHSD1 enzyme comes in 2 functional forms that differ by a single missense-encoding germline variation. The more common adrenal-restrictive allele encodes for a protein that undergoes more rapid protein degradation and results in slow conversion from DHEA to potent androgens, that is, T and DHT. In contrast, a less common adrenal-permissive allele has a single amino acid change that substitutes a threonine in place of an asparagine, confers resistance to ubiquitin-mediated enzyme degradation, increases conversion of DHEA to potent androgens, thus leading to greater stimulation of AR from nongonadal androgens (25, 39). The effectively more active adrenal-permissive allele is in the germline at about a 30% to 40% allele frequency in people of European genetic ancestry and about 5% to 10% in people of African and East Asian genetic ancestry (25).

Germline inheritance of the adrenal-permissive *HSD3B1* allele has clinical consequences on prostate cancer outcomes. From a mechanistic standpoint, an increase in the conversion from DHEA (or other nongonadal precursor steroids) to potent androgens by adrenal-permissive inheritance would be expected to enable AR stimulation during ADT and the absence of gonadal T. In men with prostate cancer who have biochemical recurrence or low-volume metastatic disease, inheritance of a single adrenal-permissive *HSD3B1* allele is associated with more rapid progression and decreased survival after ADT (25, 31, 40). Furthermore, a study of more than 5000 men with prostate cancer shows that *HSD3B1* inheritance is the most common monogenic link to prostate cancer mortality (41, 42). However, the therapeutic actionability of these clinical studies has yet to be realized. There are at least 2 approaches for pharmacologic targeting of 3βHSD1, which include direct enzymatic inhibition and pharmacologic blockade of the BMX kinase, which is required for 3βHSD1 metabolic function (43, 44). Both approaches await clinical testing.

## Biochemical clues to abiraterone resistance

CYP17A1 inhibition with abiraterone has been an overwhelmingly successful clinical strategy and is now integrated into the standard of clinical care worldwide for prostate cancer in many disease states. The essential idea of CYP17A1 inhibition for blockade of androgen biosynthesis is based on human genetic evidence that an intact CYP17A1 is necessary to sustain androgens for developmental and physiologic processes (45, 46). However, several layers of biochemical and clinical data have shown that androgen biosynthesis persists during ADT + CYP17A1 inhibition. The clinical evidence for persistent androgens includes mass spectrometry analyses for residual urinary androgens (47) and prostate tissue androgens from neoadjuvant clinical trials of CYP17A1 blockade with abiraterone (48). While it is possible that abiraterone incompletely blocks CYP17A1, other pharmacologic inhibitors that were subsequently designed and clinically developed never showed any improvement in clinical outcomes compared with abiraterone (49). Together, it appears that CYP17A1 inhibition does not completely suppress androgen biosynthesis.

### CYP11A1

An alternative to CYP17A1 blockade is to go upstream for pharmacologic blockade of CYP11A1, which is the very first enzyme required to convert cholesterol to androgens, as well as the biosynthesis of all other steroids. This strategy has been pursued with MK-5684 (formerly called ODM-208), which has moved from preclinical to clinical development and now into 2 worldwide randomized phase 3 clinical trials in prostate cancer (10, 50). To date, CYP11A1 inhibition appears to be most active in men with metastatic CRPC who have detectable mutations in the AR ligand-binding domain (51). This observation is consistent with the clinical activity of CYP11A1 inhibition being largely attributable to blocking the biosynthesis of nonandrogen steroids that aberrantly stimulate the mutated AR, as opposed to T and/or DHT stimulating the wild-type AR. The other notable item for clinical development and interrogation of clinical activity of CYP11A1 inhibition is that replacement of other steroids with glucocorticoid and mineralocorticoid activity is necessary in order to prevent adrenal insufficiency.

### CYP17A1- and CYP11A1-independent androgen biosynthesis

Could there be enzymes and pathways for androgen biosynthesis that circumvent the 2 known cytochrome p450 enzymes that are thought to be absolutely necessary? Human-derived cell line models of CRPC generate physiologically relevant concentrations of intracellular T and DHT when cultured in the absence of serum, as well as in the presence of CYP17A1 pharmacologic inhibitors, thus raising this very intriguing possibility that a CYP17A1-independent pathway exists, which circumvents the enzymatic step that is blocked by abiraterone (52). Treatment with ^13^C-cholesterol in the same cell culture systems led to the generation of ^13^C-testosterone which further demonstrates that prostate cancer has the cellular machinery to autonomously convert cholesterol to androgens in the absence of an exogenous endocrine organ source for precursors.

**Figure 2:**
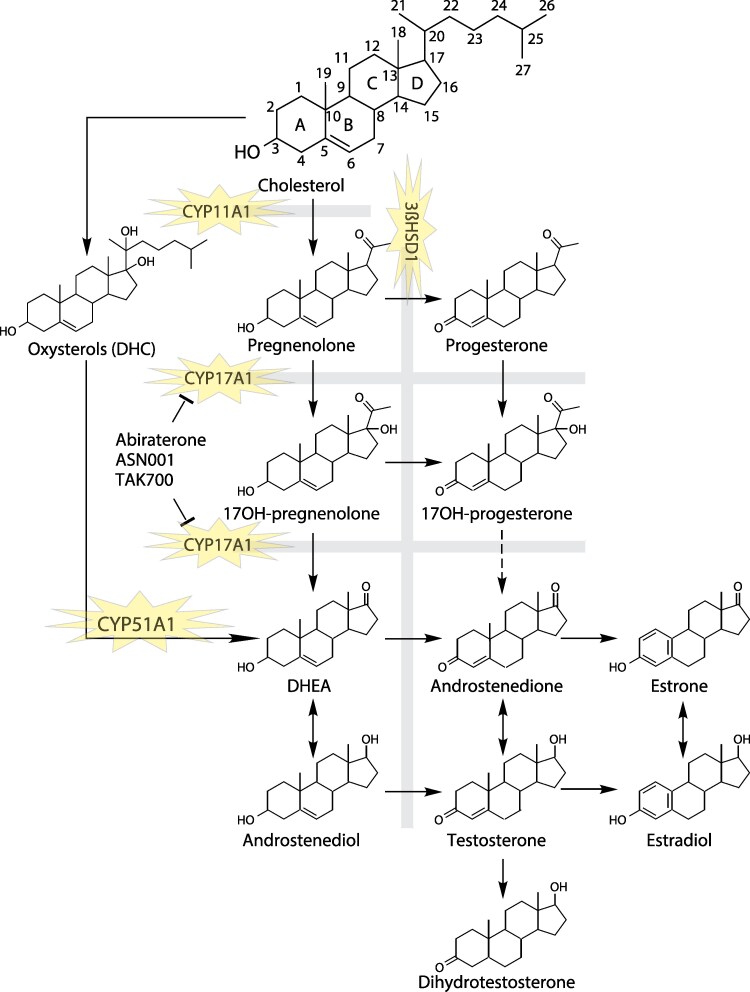
A CYP51A1-dependent pathway from cholesterol circumvents both CYP17A1 and CYP11A1 for androgen and estrogen biosynthesis. From Zhu, et al *Nature Comm* 2025.

Oxysterols are oxidized forms of cholesterol that are generally not thought to be involved in steroid biosynthesis (53). However, some oxysterols are noted to stimulate the activity of the sex steroid receptors, which is generally thought to be independent of their further metabolism (54). Surprisingly, a specific dihydroxylated oxysterol was recently found to be a substrate for androgen biosynthesis. Oxysterol conversion to androgens was analytically confirmed with deuterium-labeling studies. Furthermore, an unbiased approach to functional expression and evaluation of all human p450 enzymes showed that only CYP51A1 is capable of androgen biosynthesis from oxysterols (Fig. 2). Notably, neither CYP17A1 nor CYP11A1 can replace this previously unappreciated androgen biosynthesis activity of CYP51A1 using oxysterol substrates (52). The clinical consequences of these surprising observations have yet to be determined.

### Gut microbiome and androgen metabolism

In addition to the steroid metabolic activities that occur in human cells, there is substantial interest in understanding the role of gut and other microbial enzymes in steroid metabolism and hormone therapy resistance. This is an incredibly complex area, as it would require an understanding of metabolic activities of a complex array of microbes that dynamically change over time with various dietary, environmental, and treatment exposures. Microbial composition in physiologic environments is often made up of a complex array microbial species. Therefore, metagenomic data are frequently used to infer the metabolic activities associated with enrichment or depletion of specific bacterial species (55). Furthermore, certain bacterial enzymes are reported to have various steroid-metabolizing activities that include conversion of 21-carbon steroids to androgens, for example, conversion of cortisol to 11β-OH-A4 (56). Such microbial enzymes are often not susceptible to pharmacologic inhibition, as is the case with human enzymes that perform comparable reactions for the generation of androgens. Similar to studies and approaches with human enzymes, studies with microbial species require direct biochemical and analytical observations for definitive identification of reactions and steroid species (56).

Additional considerations related to gut microbiome steroid metabolism include the physiology that regulates the interface between the gut intraluminal compartment and blood. For example, bile acids, which are also made from cholesterol, are synthesized in the liver, enter the small intestine, and after digestive processes, they are actively transported out of the luminal space into blood, being recycled back to the liver (57). Approximately 95% of bile acids undergo enterohepatic recycling (58). For the steroid metabolic products of the gut microbiome to affect prostate cancer tumors, the metabolites must find a way to get back into circulation, whether it be through passive diffusion, enterohepatic circulation, or some other process. Here, it should also be noted that enterohepatic cycling would necessitate first-pass metabolism in the liver, which is known to have major steroid-metabolizing activities, including as a major site of expression for SRD5A1 and AKR1D1, the latter of which encodes a 5β-reductase that generally inactivates steroids (59). Thus, an understanding of how gut-derived steroids are processed in the liver is necessary to determine what prostate cancer could be exposed to.

## Conclusions

The evolution of hormonal therapies for prostate cancer has developed from an emphasis on gonadal androgens to nongonadal androgens that necessarily have to undergo metabolic conversion in various physiologic compartments (adrenal, prostate cancer tissue) to potent endogenously produced androgens. CYP17A1 inhibition as an approach for nongonadal androgen synthesis inhibition has been extensively exploited as a therapeutic strategy. However, the heterogeneity of clinical response, the role of genetics in treatment responsiveness and absence of complete androgen synthesis inhibition with CYP17A1 inhibition, all beg for pressing the exploration of alternative metabolic enzymes as targets. CYP11A1 inhibition has entered into large-scale randomized clinical trials. Other metabolic targets that have emerged include 3β-HSD1, AKR1C3, and CYP51A1. One of the major challenges that accompanies clinical testing of these new hormonal therapy strategies is the identification of the correct clinical context for early phase clinical trials. For example, the identification and development of mechanistically based biomarkers that would identify responders in phase 1/2 clinical trials would enable successful clinical development, where heterogeneity inevitably exists that would otherwise impede clinical development. A refined perspective on clinical evaluation and clinical trials will be essential for the appropriate development of these new therapeutic strategies for prostate cancer (60).

## Disclosures

N.S. is a co-inventor on Cleveland Clinic patent applications on *HSD3B1.*

## Data Availability

There are no original data in this mini-review.

## Abbreviations

3βHSD1: 3β-hydroxysteroid dehydrogenase 1
AD: Δ^4^-androstenedione
ADT: androgen deprivation therapy
AR: androgen receptor
ARSI: androgen receptor signaling inhibitor
CRPC: castration-resistant prostate cancer
DHEA: dehydroepiandrosterone
DHT: 5α-dihydrotestosterone
SRD5A: steroid-5α-reductase
T: testosterone
